# Testicular prostheses in patients with testicular cancer - acceptance rate and patient satisfaction

**DOI:** 10.1186/s12894-015-0010-0

**Published:** 2015-03-13

**Authors:** Klaus-Peter Dieckmann, Petra Anheuser, Stefan Schmidt, Benjamin Soyka-Hundt, Uwe Pichlmeier, Philipp Schriefer, Cord Matthies, Michael Hartmann, Christian G Ruf

**Affiliations:** Department of Urology, Albertinen-Krankenhaus Hamburg, Hamburg, Germany; Department of Urology, Bundeswehrkrankenhaus Hamburg, Hamburg, Germany; Institute of Medical Biometry and Epidemiology, Universitätsklinikum Eppendorf, Hamburg, Germany; Department of Urology, Universitätsklinikum Eppendorf, Hamburg, Germany

**Keywords:** Testicular cancer, Testicular prosthesis, Orchiectomy, Masculinity, Quality of life, Body appearance

## Abstract

**Background:**

The loss of a testicle to cancer involves much emotional impact to young males. Little is known about the number of patients with testicular germ cell tumour (GCT) who would accept a testicular prosthesis. Also, knowledge about the satisfaction of implant recipients with the device is limited.

**Methods:**

A retrospective chart analysis was performed on 475 consecutive GCT patients. Prior to orchiectomy, all patients were offered prosthesis insertion. Acceptance of implant was noted along with age, clinical stage, histology and year of surgery. 171 implant recipients were interviewed using an 18 item questionnaire to analyze satisfaction with the prosthesis. Statistical analysis involved calculating proportions and 95% confidence intervals. Multivariate analysis was performed to look for interrelations between the various items of satisfaction with the implant.

**Results:**

26.9% of the patients accepted a prosthesis. The acceptance rate was significantly higher in younger men. Over-all satisfaction with the implant was “very high” and “high” in 31.1% and 52.4%, respectively. 86% would decide again to have a prosthesis. Particular items of dis-satisfaction were: implant too firm (52.4%), shape inconvenient (15.4%), implant too small (23.8%), position too high (30.3%). Living with a permanent partner had no influence on patient ratings. Multivariate analysis disclosed numerous inter-relations between the particular items of satisfaction.

**Conclusions:**

More than one quarter of GCT patients wish to have a testicular prosthesis. Over-all satisfaction with implants is high in more than 80% of patients. Thus, all patients undergoing surgery for GCT should be offered a testicular prosthesis. However, surgeons should be aware of specific items of dis-satisfaction, particularly shape, size and consistency of the implant and inconvenient high position of the implant within the scrotum. Appropriate preoperative counselling is paramount.

**Electronic supplementary material:**

The online version of this article (doi:10.1186/s12894-015-0010-0) contains supplementary material, which is available to authorized users.

## Background

The loss of a testicle due to cancer has considerable impact on the sexual life and over-all quality of life in survivors of patients with testicular germ cell tumour (GCT) because this is felt to be a threat to masculinity by many patients [1]. That loss is associated with feelings of uneasiness or shame about impaired body appearance in one quarter of the patients and roughly one third of GCT patients do actually miss or have previously missed their lost testicle [2]. Not surprisingly, younger men perceive the loss of a testicle more often a humiliating situation than older men do [3]. From a practical point of view, replacement of a testicle by a testicular prosthesis is technically simple, and only few surgical complications are to be expected [4,5]. Generally, questions surrounding the quality of life have increasingly gained attention among physicians caring for GCT patients [6]. However surprisingly, despite the ever increasing total number of reports relating to GCT, the issue of testicular implants has been addressed only sporadically. The first testicular prosthesis was implanted in 1941 [7]. Technical refinements regarding the material of the device were reported subsequently [8] until the silicone-made testicular prosthesis was introduced in 1973 [9]. That type of implant is still in use with only few modifications made [10,11]. There are some reports on the technical feasibility of testicular prosthesis insertion and on surgical problems relating to this procedure [12-15], but very few studies have systematically explored the patient view on testicular implants. In particular, little information exists as to how many patients would accept an implant in the case of orchiectomy for GCT and how those having received such a device are satisfied with it. Remarkably, none of the current international guidelines on treatment of GCT address the option of prosthesis implantation subsequent to orchiectomy [16-19]. We retrospectively looked to our sample of testis cancer patients to find out how many of them accepted an implant and if there were any associations with age and oncological characteristics. Further, we asked recipients of testicular prostheses about their satisfaction with the implant using a questionnaire.

## Methods

Since 1997, it was the policy of our department to offer the implantation of a testicular prosthesis to patients undergoing surgery for GCT and who were not older than 60 years. From January 1997 to June 2014 a total of 507 patients underwent inguinal orchiectomy for testicular cancer. We retrospectively analyzed the patient files and noted whether or not the patient had been offered and if so whether they had accepted a testicular prosthesis. To look for any association of prosthesis acceptance with clinical characteristics, the following parameters were registered additionally: patient’s age, histology of GCT, and clinical stage. To look for any temporal association of implant acceptance, the year of orchiectomy was noted. 475 patients (293 pure seminoma, 183 nonseminoma) qualified for further analysis.

The patient perception of living with a testicular implant was studied by interviewing the recipients with a structured questionnaire after obtaining written informed consent from the patients. Only adult patients were asked to participate in the study. To obtain a sufficient number of patients for a meaningful statistical analysis, the questionnaire was sent to implant recipients of three Hamburg based testicular cancer units (Albertinen-Krankenhaus, Bundeswehr-Krankenhaus, Universitätsklinikum Eppendorf). Candidates for interview were defined by the diagnosis of unilateral testicular germ cell tumour and by having the implant for at least half a year and no longer than 10 years. The response rate was 41% and a total of 171 questionnaires were available for analysis. The questionnaire involved 18 questions (see Additional file 1) with multiple choice answers and one question with free-text answering. The study had been approved by the institutional ethical committee of the Theologisches Seminar Elstal (Wustermark).

### Statistical analysis

The data of both parts of the study were filed in a commercially available system (Microsoft Excel) prior to further analysis. The final statistical analysis was accomplished by using descriptive statistical methods performed with the SAS software package (version 9.3, SAS Institute, Inc., Cary, NmC, USA) on Windows platform. To derive exact confidence intervals for multinominal parameters, StatXact (Version 9.0, Cytel Software Corporation) was applied. Pre-defined hypotheses were subjected to statistical analysis. For nominal variables, Chi-square tests were applied. Exact Cochran-Armitage trend tests were employed for testing ordered binominal populations and the exact Jonckhere Terpstra tests for doubly ordered contingency tables, respectively. Ordinal variables between two groups were tested using Mann-Whitney tests. For multivariable assessment of the probability of acceptance logistic regression models were derived [20].

## Results

A total of 128 patients of the unselected GCT cohort accepted a testicular prosthesis (26.9%). Tables 1 and 2 show the acceptance rates in subgroups and the associations with clinical parameters. The acceptance rate did not change over time. The only significant association was with age (Cochran Armitage Trend Test p = 0.0058). The acceptance rate was 30.5% in patients younger than 40 years while it was 19.3% in the older age group. The parameter “age” remained almost significant (p = 0.0503) upon multivariate analysis.

**Table 1:** **Acceptance rate of testicular prostheses in unselected cohort of GCT patients**

	**Total**	**With prosthesis**	
	**n (%)**	**n (%)**	**95% CI**
**Total sample**	475 (100)	128 (26.9)	23.0%; 31.2%
Histology*			
Seminoma	293 (100)	81 (27.6)	22.6%; 33.1%
Nonseminoma	182 (100)	47 (25.8)	19.6%; 32.8%
Clinical stage**			
CS1	313 (100)	91 (29.1)	24.1%; 24.4%
CS 2	138 (100)	32 (23.2)	16.4%; 31.1%
CS 3	24 (100)	5 (20.8)	7.1%; 42.2%
**Age at diagnosis** ^§^			
≤20 years	16 (100)	4 (25.0)	7.3%; 52.2%
>20 - ≤ 30 years	103 (100)	34 (33.0)	24.1%; 43.0%
>30 - ≤ 40 years	206 (100)	61 (29.6)	23.5%; 36.4%
>40 - ≤ 50 years	113 (100)	25 (22.1)	14.9%; 30.9%
>50 - ≤ 60 years	37 (100)	4 (10.8)	3.0%; 25.4%
**Treatment** ^#^			
1997 - 2003	143 (100)	35 (24.5)	17.7%; 32.4%
2004 – 2009	167 (100)	55 (32.9)	25.9%; 40.6%
2010 – 2014	165 (100)	38 (23.0)	16.8%; 30.2%

*histology: chi-square test p = 0.66.

**clinical stage: Cochran-Armitage Trend Test p = 0.07.

^§^age at diagnosis: Cochran-Armitage Trend Test p = 0.006, significant.

^#^episode of treatment: Cochran-Armitage Trend Test p = 0.38.

CI 95% confidence intervals.

**Table 2:** **Acceptance of prosthesis – logistic regression model**

**Parameter**	**Odds ratio**	**95% Wald CI**		p-value*
**Age (years)**				0.05
≤20 vs. > 30 - ≤ 40	1.06	0.31	3.62	
>20 - ≤ 30 vs. > 30 - ≤ 40	1.28	0.75	2.17	
>40 - ≤ 50 vs. > 30 - ≤ 40	0.64	0.37	1.11	
>50 - ≤ 60 vs. > 30 - ≤ 40	0.28	0.09	0.82	
**Histology**				0.50
Seminoma yes vs. no	0.84	0.52	1.38	
**Clinical stage**				0.33
CS2 vs. CS1	0.71	0.43	1.15	
CS3 vs. CS1	0.70	0.23	1.93	
**Year of treatment**				0.09
2004 - 09 vs. 1997 – 2003	1.65	0.99	2.74	
2010-14 vs. 1997 – 2003	1.06	0.61	1.82	

*Wald chi square; CS clinical stage; CI confidence intervals.

The statistical analysis of the questionnaire was somewhat hampered by the fact that some of the 171 patients did not answer to every question. So, sample size varies with each question (Table 3). The majority of implant recipients (77.4%) were living with a permanent partner and the majority had the device for more than 2 years (74.3%). 4.8% required additional surgery. To “look normal with regard to the genital region” was rated “extremely important” or “important” in 53% and 32% of patients, respectively. Accordingly, 98% of the responders regarded the preoperative offer of an implant important. However, preoperative counseling with respect to testicular prosthesis insertion was valued “too short” by 31% and even “insufficient” by 8.5%. With respect to the physical appearance of the implant, 52.4% valued the consistency “too firm” and 15.4% rated the shape of the implant “not convenient” with most of the dissatisfied men saying it was “too round”. 9.8% sensed the implant as a foreign body. Size of the implant was regarded “too small” by 23.8% while 30.3% criticized a “too high” position of the implant within the scrotum.

**Table 3:** **Results of questionnaire regarding patients’ satisfaction with testicular implant**

**Question**	**Eligible (n)**	**Answer**	**(%)**	**95% CI**
Married	167	yes	50.3	41.4%; 59.2%
Living with permanent partner	164	yes	77.4	69.5%; 84.0%
Time living with implant	168	<1 year- 2 years > 2 years	14.9	9.3%; 22.3%
			10.7	5.8%; 17.4%
			74.4	65.7%; 81.8%
Additional surgery after implant insertion	170	yes	4.7	2.1%; 9.6%
Normal appearance with two testicles: important?	168	very important	53.6%	44.1%; 62.6%
		important	31.3	23.2%; 40.6%
Being offered a prosthesis, preoperatively	169	important	98.2	94.2%; 99.6%
Size of the implant	164	too large	9.8	5.0%; 16.3%
		too small	23.8	16.3%; 32.3%
Weight of the implant	166	too heavy	6.6	2.9%; 12.5%
		Just right	91	84.4%; 95.2%
Shape of implant	169	not right	15.4	9.7%; 22.5%
Consistence of implant	164	too firm	52.4	43.4%; 61.1%
Position of implant within scrotum	165	too high	30.3	22.0%; 39.6%
Any particular feeling with the implant	164	convenient	16.5	10.0%; 24.6%
		inconvenient	4.3	1.5%; 9.7%
		strange	9.1	4.6%; 16.0%
Problems with implant during physical exercise	164	no	92.1	85.8%; 96.1%
Concerns about future problems with the implant	164	no	89.0	82.7%; 93.6%
Counseling before implant placement	164	too short	31.1	22.7%; 40.5%
		Insufficient	8.5	4.2%: 14.9%
After all, would you have an implant again?	165	yes	86.1	79.1%; 91.4%
Over-all satisfied with implant	164	very well	31.1	22.2%; 41.0%
		satisfied	52.4	42.1%; 62.4%
		just so	12.2	6.5%; 20.0%
		no	4.3	7.4%; 1.2%

Despite dissatisfaction with several particular items, the over-all satisfaction with the implant was very high and high in 31.1% and 52.4%, respectively. Accordingly, 86.1% would opt again for receiving an implant in the case of orchiectomy.

Living with or without a permanent partner had no influence on any of the patient ratings. However, multivariate analysis disclosed several significant associations between the physical attributes of the implant (Table 4). Inappropriate size of the implant was associated with perceived excess weight of the device and with inconvenient shape, respectively. Inappropriate position within the scrotum correlated with insufficient weight of prosthesis. The appraisal “too firm” was associated with inconvenient shape and with inappropriate position. Over-all satisfaction was significantly higher in patients having the prosthesis for more than two years (p = 0.015; exact Jonckhere-Terpstra Test). Over- all satisfaction was 54.5% in patients who required additional treatment, while it was 85.6% among those without such procedures (p = <0.001, exact Mann-Whitney Test). Likewise, 87.3% of the patients opting again for a prosthesis were (very well and well) satisfied while only 60.8% deciding against redoing the implant were so. Over-all satisfaction was significantly associated with appropriate size of the implant, as well as with shape and consistency (Table 5).

**Table 4:** **Significant associations between various items of satisfaction with prosthesis**

	**Size of prosthesis**			
	**Too much**	**Just right**	**Too small**	
**Weight of prosthesis**				p = 0.032*
Too heavy	18.8%	4.6%	7.9%	
Right	81.3%	95.4%	81.6%	
Too light	-	-	10.5%	
**Shape of prosthesis**			p = 0.002^#^	
Inconvenient	31.3%	8.3%	28.2%	
Just right	68.8%	91.7%	71.8%	
	**weight of prosthesis**			
**Position within scrotum**				p = 0.023^§^
Right	81.8%	69.6%	-	
Not right	18.2%	30.4%	100%	
	**shape of prosthesis**			
**Consistency of prosthesis**			p = 0.017^#^	
Too firm	75%	48.6%		
Convenient	25%	51.4%		
	**consistency of prosthesis**			
**Position of prosthesis**			p = 0.035*	
Right	61.6%	76.9%		
Too high	38.4%	23.1%		

*Exact Jonckhere Terpstra test.

^#^chi square test.

^§^Exact Cochran Armitage Trend Test.

**Table 5:** **Significant associations of over-all satisfaction with particular items of patients’ satisfaction**

	**Over-all satisfaction with testicular implant**					
	**Very well**	**Well**	**Just so**	**Not much**	**Not at all**	
**Size**						p < 0.001*
Too large	7.8%	13.3%	-	-	33%	
Right	82.4%	65.1%	31.6%	100%	-	
Too small	9.8%	21.7%	68.4%	-	66.6%	
**Shape**						p = 0.001**
Inconvenient	5.9%	14.0%	35.0%	-	66.7%	
Convenient	94.1%	86.0%	65.0%	100%	33.3%	
**Consistence**						p < 0.001**
Too firm	20.0%	62.8%	75.0%	100%	100%	
Convenient	80.0%	37.2%	25.0%	-	-	

All numbers represent percentages, *Jonckhere-Terpstra Test, **Cochran-Armitage Trend Test.

## Discussion

Surprisingly few studies have so far explored patient attitudes to receiving a prosthesis to replace a testicle that has been lost to cancer. It is thus valuable to note that more than one quarter of GCT patients (26.9%) in the present series decided to have such a device. Expectedly, testicular implants are more frequently requested by younger patients. This observation has already been made previously [15,21,22] and it is also in close accordance with the experience that body appearance is of greater importance to younger men [3].

Adshead et al. noted lower acceptance rates in married men and in those living in steady relationship [23]. We could not directly confirm this observation because we did not look to the marital status of the patients in our retrospective analysis. The inverse association of steady relationship and prosthesis acceptance appears probable as older men are more frequently married or live in steady relationship than younger men.

The likelihood of accepting a prosthesis decreases with age and likewise with the probability of living in steady relationship. Clinical stage and histology of the GCT did not influence the decision to have an implant in the present study.

The acceptance rate of 26% in our series is somewhat lower than the rates of 55% [15], 46% [24], and 43% [25] reported earlier. Our report is in line with the 24% rate found in a large and unselected GCT population from Swedish hospitals [3] and it is not significantly different from the rate of 30% reported in a British series [23]. The reasons for the large differences regarding the acceptance rates among the reported series remain elusive. Selection in favour of young patients [24] could represent one possible bias, and chance due to small sample size [25] another. A patient’s wish to have a prosthesis is a complex decision [26]. As documented in a Swedish survey on survivors of GCT, 32% of patients reported feelings of loss and uneasiness or even shame secondary to the excision of a testicle. Acceptance rates of testicular prostheses are in close accordance with the prevalence of feelings of reduced masculinity secondary to orchiectomy.

As revealed in the second part of the present study, preoperative counselling is highly valued by the patients. Thus, the way and extent of professional advice prior to orchiectomy for TC will probably represent one cornerstone in patient decision-making regarding testicular implants [27].

In contrast to other reports, we did not find a trend to higher acceptance rates in recent years [3,24].

**Figure 1:**
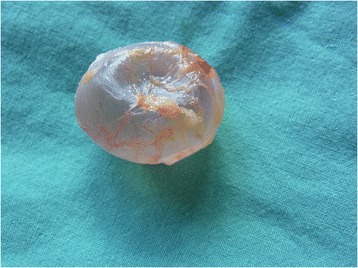
**Silicone testicular prosthesis removed from scrotum because of shrinkage: note prune-like appearance of the device.**

With regard to surgical complications, 4.7% of our patients required additional surgery. This result is well in line with the previously reported incidence of surgical complications of 2.6% to 8% following testicular prosthesis insertion [4,14,15,28-30] and noteworthy, it is not higher than the 8% complication rate encountered after inguinal orchiectomy [31]. We did not experience ruptures of prosthesis or spontaneous extrusion but we did replace three devices because of shrinkage (Figure 1).

The over-all aesthetic results of testicular implants are far from ideal and this is probably the most important result of the present study. Dissatisfaction mostly relates to consistency (too firm), inappropriate size (too small), shape (too round), and to the position within the scrotum (too high). As revealed by multivariate analysis, there are numerous cross-associations between these items of satisfaction. Noteworthy, most of the previous studies reported very similar results (Table 6) [21,23,24,28,32,33]. Only one study from France revealed a somewhat higher degree of satisfaction with the particular aesthetic results [34]. The reasons for unfavourable aesthetic outcome are probably three-fold: iatrogenic (i.e. physician-made), manufacturer-made, and nature-related. Consistency (too firm) and shape (too round) are probably related to technical and economic aspects of the manufacturing process but (hopefully), improvement should be possible as soon as manufacturing companies acknowledge the problem. Inappropriate size of implants appears to be an avoidable problem because it is the surgeon’s duty to insert the best-fitting prosthesis intra-operatively. A 33% dissatisfaction rate regarding the size (too small and too large) of the implants as noted in our series is rather surprising because, it is quite simple to select the appropriate size of the device from three or four available sizes. Yet, four previous studies also reported a very similar degree of dissatisfaction with size indicating that this kind of criticism is not uncommon. Dissatisfaction with size is much influenced by the patient’s emotional appraisal of the implant. It is likely that dissatisfaction with prosthetic size is based on both, surgeon-related and patient-related misconceptions. To minimize such discontent, patients should be invited to actively participate in preoperative decision-making upon implant-size.

**Table 6:** **Survey of the literature – patients’ satisfaction with testicular implants**

**Author**	**Year**	**Country**	**(n)**	**Method**	**Over-all satisfaction**	**Have it again**	**Shape**	**Position**	**Size**	**Consistency**	**Other**
Petersen [24]	1992	Ger	119	I	93%	93%	-	high 11%	19% too large	10% too firm	-
									14% too small		
Lynch [32]	1992	UK	19	Q	79%	95%	-	-	-	-	−
Incrocci [33]	2001	NL	22	Q	95%	95%	-	-	-	29% inconvenient	–
Adshead [22]	2001	UK	71	Q	73%	90%	32% not right	27% not right	37% inconvenient	-	30% weight inconvenient
Boy [28]	2002	Ger	39	Q	97%	-	-	3% too high	36% not right	3% too firm	partners’ rating: 55% satisfied
Xylinas [34]	2008	F	63	Q	96%	96%	12% not right	3% not right	5% too small	12% too firm	2% too cold
Yossepowitch [21]	2011	Isr	86	I	88%	86%	-	39% too high	27% not right	73% too firm	after 2005 better results

I interview; Q questionnaire.

The inconvenient position of many of the implants (mostly too high within the scrotum) is probably related to two reasons. First, it could be a surgical failure to create a scrotal pouch not large enough to host the implant. The proper surgical procedures have been extensively reviewed [24,35]. However, inescapable biological processes i.e. tissue reactions to the synthetic material introduced into the scrotal cavity do possibly account for shrinkage of the scrotal wall and thus cause upward migration of the prosthesis. Analogous scarring reactions are known from breast implants [36]. It is thus paramount for the surgeon to both, employ the appropriate surgical technique to ensure the right position of the implant and to advise the patient preoperatively about biological processes that may cause inadvertent high position. Noteworthy, inappropriate high scrotal position of implants has been observed in 27-39% by two previous studies [21,23]. Positioning of the prosthesis appears to be a major problem and every surgeon performing such operations should be aware of the issue [37].

The answers to the more general questions of the questionnaire indicated a high rate of over-all contentment with the implant. No more than 10% of patients are concerned about potential health problems originating from the implant. Only few patients complained of inconvenient feelings with the implant (3.7%). Some bother with the device upon physical activity was reported by 8.6% which is less than the 15% rate reported previously [21]. Accordingly, the over-all satisfaction rate is 83% (very high and high satisfaction). But noteworthy, over-all satisfaction is significantly influenced by contentment with the particular items of size, shape and consistency of the implant.

86% of patients would decide again to have a prosthesis. This rate is identical to the result reported by Yossepowitch et al. [21] and it is similar to other studies reporting rates of slightly more than 90% [23,24,28,32-34] (Table 6).

Limitations of our study include the lack of a control group of patients who declined the offer of a prosthesis. Exploring the particular reasons for deciding against a prosthesis could aid in obtaining a clearer image of young men’s emotions when they are confronted with the necessity of losing a testicle to cancer. Another drawback could be the lack of information regarding the manufacturer of the prostheses. Thus, relating specific items of dissatisfaction to the products of particular companies is not possible. On the other hand, strengths of our investigation include, first, the multicentric method of obtaining data on patients’ perception of testicular implants and second, the investigation of the largest patient sample studied for this purpose to date. The latter two issues probably aid to keep selection bias low. The employment of multivariate statistical methods to reveal cross-associations of various items of satisfaction with implants is perhaps another strength of the present study.

## Conclusions

More than one quarter of all testis cancer patients wish to receive a prosthesis to replace the excised testicle. Over-all satisfaction with the testicular implant is very high. All patients undergoing surgery for testis cancer should be advised about the availability of a testicular implant. However, it must be noted that there is considerable dissatisfaction with several particular attributes of the implants, e.g. shape, consistency, size, and high intrascrotal position. Urologic surgeons performing prosthesis insertion should be aware of these issues. Appropriate preoperative counselling with participation of the patient upon selection of implant size is paramount.

## Additional file

Additional file 1:
**Questionnaire.**
