# Supplementary material for: Testicular prostheses in patients with testicular cancer - acceptance rate and patient satisfaction
Source: BMC Urol. 2015 Mar 13;15:16. doi: 10.1186/s12894-015-0010-0 (PMC4363351; doi:10.1186/s12894-015-0010-0)
Supplement: Additional file 1: — Questionnaire. [file 12894_2015_10_MOESM1_ESM.docx]

**Additional file 1**

Questionnaire

1. Are you married?
2. Are you living with a permanent partner?
3. How long are you having your implant?
4. Did you require any medical procedures to achieve a safe healing process after implantation?
5. Did you require any surgery to correct or remove the implant?
6. Is it important for you to look “normal” with regard to the genital region?
7. Is it important for you to be offered a testicular implants before testicular surgery?
8. Is the size of your implant appropriate?
9. Is the weight of the implant appropriate?
10. Are you satisfied with the shape of your implant?
11. Is the consistency of your implant appropriate?
12. Is the position of your implant appropriate?
13. What is your feeling with the implant?
14. Do you have problems with the implant during physical exercise?
15. Are you concerned about future health problems that may arise from your implant?
16. Was the medical counselling before surgery appropriate?
17. After all, would you again decide to have an implant?
18. Over-all, are you satisfied with the implant?
19. What other problems did you have with your implant?
